# Serum Protein Electrophoresis May Be Used as a Screening Tool for Antibody Deficiency in Children and Adolescents

**DOI:** 10.3389/fimmu.2021.712637

**Published:** 2021-08-23

**Authors:** Cristina Frias Sartorelli de Toledo Piza, Carolina Sanchez Aranda, Dirceu Solé, Stephen Jolles, Antonio Condino-Neto

**Affiliations:** ^1^Department of Immunology, São Leopoldo Mandic Medical School, Campinas, Brazil; ^2^Division of Allergy, Immunology and Rheumatology, Department of Pediatrics, Federal University of São Paulo, São Paulo, Brazil; ^3^Immunodeficiency Centre for Wales, University Hospital of Wales, Cardiff, United Kingdom; ^4^Department of Immunology, Institute of Biomedical Sciences, University of São Paulo, São Paulo, Brazil

**Keywords:** antibody deficiency, calculated globulin (CG), gamma globulin fraction, children, immunoglobulin G (IgG), serum protein electrophoresis (SEP)

## Abstract

**Background:**

Patients with antibody deficiency may experience exceptionally long diagnostic delays, increasing the risk of life-threatening infections, end-organ damage, mortality, and health costs.

**Objective:**

This study aimed to analyze serum protein electrophoresis and verify the correlation between calculated globulin (CG, total protein minus albumin levels) or electrophoretically determined serum gamma globulin fraction (Gamma) with IgG levels in children and adolescents under 18 years old (yo).

**Methods:**

We analyzed serum protein electrophoresis (GC or Gamma) and IgG levels from 1215 children and adolescents under 18 yo, classified into 5 age groups. We verified the correlation between CG or Gamma with serum IgG levels.

**Results:**

Serum IgG levels varied according to age groups (from 4.3 ± 2.3 g/l in children under 6 months old to 11.4 ± 3.2 g/l in adolescents in the 10-<18 yo group). CG sensitivity and specificity to detect IgG below the reference range for all patients were 93.1% and 81.8%, respectively, and varied according to age group. Gamma sensitivity and specificity for all patients were 100% and 87.8%, respectively, and varied according to age group as well. We found serum IgG levels below the age reference level in 29 patients (2.4% of the cases) using CG or Gamma levels.

**Conclusion:**

Both CG and Gamma levels may be of utility as a screening tool for earlier diagnosis of antibody deficiency in children and adolescents under 18 yo.

## Introduction

Antibody deficiencies are the most commonly reported immunodeficiencies worldwide and may be either primary or secondary. Primary antibody deficiency (PAD) refers to a heterogeneous group of genetic disorders characterized by an intrinsic impairment in antibody production or function (1).

Inborn errors of immunity (also known as Primary Immune Deficiencies – PIDs) are a group of more than 400 diseases caused by monogenic germline mutations and characterized by increased susceptibility to infectious diseases, autoimmunity, autoinflammation, allergy, and malignancy (2). While on a global scale the commonest causes of secondary immunodeficiency include HIV and malnutrition, primary antibody deficiencies make up by far the largest subset of inborn errors of immunity including both (3) predominantly antibody deficiencies or in categories associated with defects in innate immune cells or T cells (4). Taken together, antibody deficiencies are present in 70-80% of all PIDs (5) and are recognized to be both under-diagnosed and under-reported in a systematic review of PID registries (6).

The diagnosis of quantitative antibody deficiency is generally straightforward using serum immunoglobulin measurement (7). However, patients frequently experience long delays before diagnosis and treatment (8–10). This diagnostic delay is often measured in years and can lead to end-organ damage (11) and decreased survival (12); while prompt and appropriate treatment decreases morbidity and mortality [reviewed by Perez et al. (13)]. Early diagnosis thus reduces health care expenses and leads to better health outcomes for patients with PIDs (14).

Screening methods that improve earlier identification of antibody deficiencies are of key importance in reducing diagnostic delay. T cell receptor excision circle (TREC) (15) or κ (kappa)-deleting excision circle (KREC) (16) methods are available for newborn screening of severe forms of PIDs but are not yet widely offered (17). While very successful in the detection of severe combined immunodeficiency (SCID) and potentially a small subset of agammaglobulinemia without B cells, these tests do not effectively detect diseases with a normal number of T and B cells and those with later onset, such as common variable immunodeficiency (CVID) (18).

A number of studies have demonstrated that calculated globulin (CG) can be used as a low-cost screening method for antibody deficiencies in adults (19, 20). CG is derived from the difference between total protein and albumin levels and can be calculated automatically, often as part of liver function tests (LFTs).

This study is the first to establish a correlation between CG, electrophoretically determined gamma globulin fraction (Gamma) and IgG levels in children and adolescents by age range in a Brazilian population sample. Unlike previous publications, we used protein electrophoresis to determine CG and Gamma, allowing us to correlate those with IgG levels in the same groups. Both yielded significant correlations with the IgG levels, showing that CG or Gamma could be used to screen for antibody deficiencies in children and adolescents.

## Materials and Methods

### Participant Details

In line with the Brazilian Ministry of Health and the Helsinki Convention’s rules and regulations participants aged from 0 to 18 years were recruited with consent from three different Allergy/Immunology clinics in São Paulo State, Brazil. Inclusion criteria were outpatients aged less than 18 years old, with clinically stable conditions, and informed consent. Exclusion criteria were age above 18 years old, unstable clinical conditions, and lack of informed consent.

All patients were referred for possible immunologic or allergic conditions. One hundred and eighty-eight had a final diagnosis of PID (8.9% of the cases) and 29 presented with antibody deficiency (2.4% of the cases). We did not include any patients with secondary immunodeficiency.

A 5mL blood sample was collected and patients were able to choose which laboratory undertook the analyses. All laboratories were accredited according to the Associação Brasileira de Normas Técnicas (ABNT NBR ISO 15189) (21), the Brazilian Society of Clinical Pathology (PALC) (22), and were contacted to determine equipment and testing methodology.

### Laboratory Measurements

IgG, IgA, IgM values were determined by immunoturbidimetry (Roche COBAS 6000, Roche Diagnostics International Ltd, CH-6343, Rotkreuz, Switzerland). IgG reference values were based on Adeli et al. (23). Serum protein electrophoresis (SEP) was performed using Hydrasys (Sebia, Paris, France) instruments and Hydragel Protein (E) gels (Sebia, Paris, France). The visualization of the gel provided qualitative analysis, while reading of the agarose gels on a Sebia reader provided protein profiles for relative quantitative analysis by Hydrasys 2 Scan (Sebia, Paris, France) scanning system. CG values were obtained by subtracting the albumin levels from total protein values. The gamma globulin fraction was directly determined by protein electrophoresis.

### Statistical Analysis

**Figure 1:**
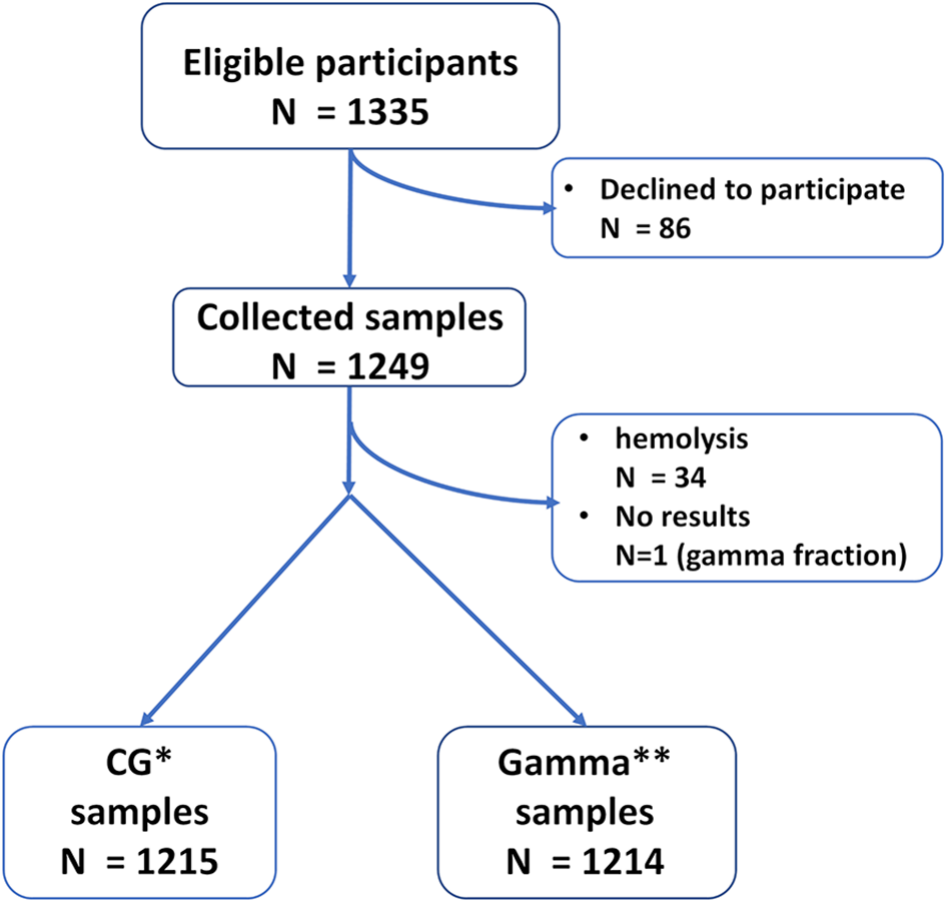
Sample flow. *CG – Calculated Globulin. **Gamma – Gamma Globulin fraction.

One thousand three-hundred thirty five (1335) consecutive patients from ages 0 to 18yo were recruited. **Figure 1** depicts the flow of excluded samples.

The Kruskal-Wallis test followed by the Mann-Whitney U test was used for IgG, CG, and Gamma levels in both studies. The Bonferroni method was used to adjust p values for multiple variables. The assumptions of normality of data distribution and homogeneity of variances were checked by the Shapiro Wilk Test and Levene Test. The chi-square test was applied to compare the frequency of occurrence between males and females in each age group. Linear regressions were performed to explore the association between IgG *vs.* CG and IgG *vs.* Gamma globulin fraction models. One-way ANOVA followed by a *post hoc* Bonferroni’s test was used to compare age groups.

The accuracy of the obtained discriminant value was interpreted based on the AUC and classified as: “perfect” (AUC = 1), “exceptional” (0.9 ≤ AUC <1), “excellent” (0.8 ≤ AUC <0.9), “acceptable” (0.7 ≤ AUC <0.8) and “poor” (AUC <0.7), noting that the AUC is not statistically different from that obtained at random for AUC values ≤ 0.5 (24). The Youden index was calculated to confirm the discriminant score, defined as the highest value observed for the following operation: sensitivity + specificity – 1 (25).

Receiver operating characteristic curves were created to identify discriminating CG and Gamma globulin cutoff values.

All analyses were conducted in PASW statistics 18.0 software (SPSS Inc., Chicago, USA), adopting a significance level (α) of 5% (P < 0.05).

## Results

The study included 1249 patients. CG analyses included 1215 samples while Gamma analyses included 1214 samples. See **Figure 1** for recruitment and sample flow details.

### Correlation Between IgG and CG Values

Descriptive data for the IgG x CG analysis are shown in **Table 1**. There was a stepwise increase observed for both IgG and CG with age.

**Table 1:** Characteristics of patients studied for the IgG *vs* CG correlation^a^.

Age Group	Age (years)	IgG (g/L)	CG (g/L)	% males
1 to 5 mos (n = 23)	0,3 ± 0,1	4,3 ± 2,3	21,1 ± 4,2	52,2
6 to 11 mos (n = 56)	0,7 ± 0,1	5,4 ± 2	23 ± 4,2	42,9
1 yo to <4 yo (n = 364)	1,8 ± 0,8	8,4 ± 3	26,8 ± 4,3	53,8
4 yoto <10 yo (n = 442)	6,3 ± 1,7	10,2 ± 3	27,9 ± 4	51,7
10 yo to <18 yo (n = 330)	13 ± 2,3	11,4 ± 3,2	29,4 ± 4,5	55,2
All (n = 1215)	6,4 ± 4,8	9,7 ± 3,4	27,6 ± 4,6	52,9

a Data are presented as mean ± SD.

In analyzing discriminant cutoff values between patients with levels below the reference and normal for IgG from CG, the predictive power was classified as excellent to exceptional (AUC from 0.91 to 0.96). AUC was significant and with acceptable accuracy for all age groups, except those younger than 1 yo (**Table 2**). For these groups, we could not establish discriminant CG cutoff values between patients with levels below the reference and normal for IgG because there were no patients with hypogammaglobulinemia. Sensitivity values ranged from 90.9% to 100.0% in the remaining age groups. The specificity values ranged from 80.2% to 94.7%. Good accuracy was also observed for the cutoff value obtained regardless of the participants’ ages (AUC = 0.916, P <0.001, sensitivity = 93.1% and specificity = 81.8%).

**Table 2:** CG values as a function of IgG levels.

Age Group	AUC	95% CI	p value	CG Cutoff value (g/L)^a^	Sensitivity	Specificity	Number of patients with IgG	
							Below reference values	Normal
1 to 5 mos (n = 23)	–	–	–	–	–	–	0	23
6 to 11 mos (n = 56)	–	–	–	–	–	–	0	56
1 yo to <4 yo (n = 364)	0,965	0,92 - 1	<0,001	23,1	1	0,838	6	358
4 yoto <10 yo (n = 442)	0,951	0,91 - 0,99	<0,001	24,8	1	0,802	12	430
10 yo to <18 yo (n = 330)	0,945	0,85 - 1	<0,001	24,1	0,909	0,947	11	319
All (n = 1215)	0,916	0,87 - 0,96	<0,001	24,1	0,931	0,818	29	1186

AUC, area under the curve. CI 95%, 95% confidence interval.

a CG values below which IgG levels were considered below reference.

**Figure 2:**
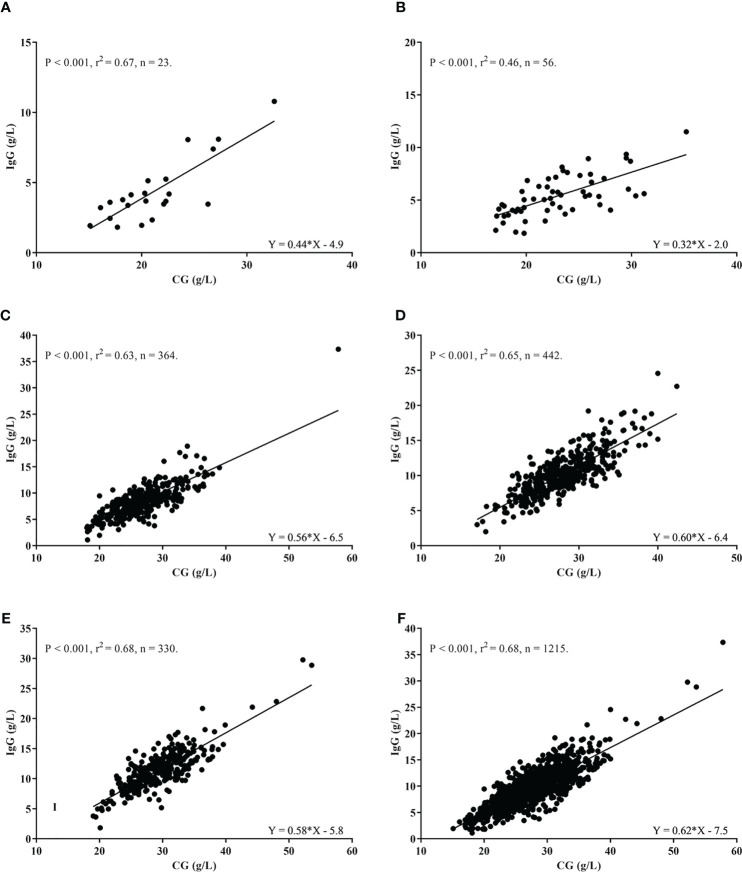
Correlation between IgG (g/L) and Calculated Globulin values (g/L) according to age groups. **(A)** 1 to 5 months. **(B)** 6 to 11 months. **(C)** 1 to 3 years. **(D)** 4 to 9 years. **(E)** 10 to <18 years. **(F)** All age groups combined.

A significant positive relationship in simple linear regression was observed between GC and IgG values for all age groups analyzed separately or in a combined analysis. CG values were able to significantly explain part of the IgG values variance for all age groups: 1 to 5 mos., 67%, (**Figure 2A**); 6 to 11mos., 46%M, (**Figure 2B**); 1 to 3 years, 63%, (**Figure 2C**); 4 to 9 years, 65%, (**Figure 2D**); 10 to <18 years, 68%, (**Figure 2E**); Additionally, when analyzing the entire cohort, CG values explained 68% of IgG % (**Figure 2F**).

### Correlation Between IgG and Electrophoretically Determined Gamma Globulin Fraction (Gamma) Fraction Values

**Figure 3:**
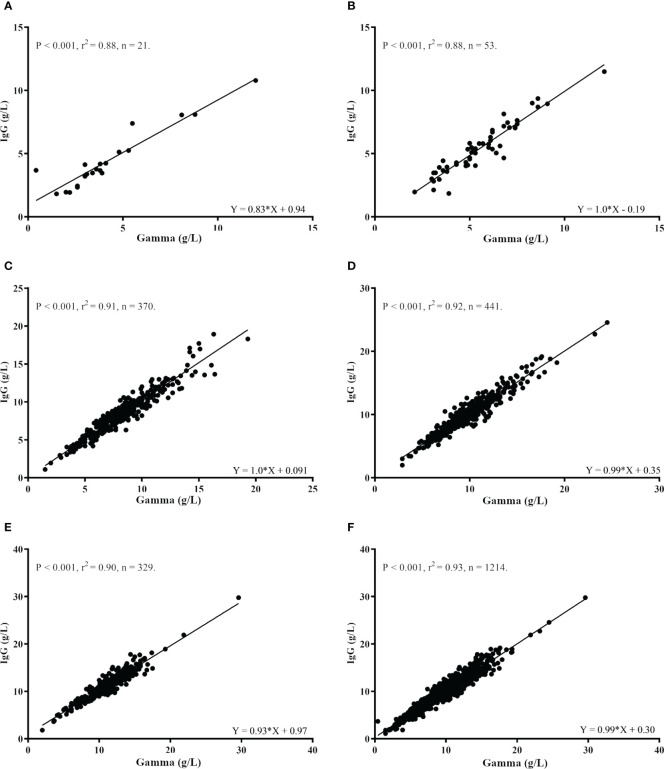
Correlation between IgG (g/L) and Gamma globulin values (g/L) according to age groups. **(A)** 1 to 5 months. **(B)** 6 to 11 months. **(C)** 1 to 3 years. **(D)** 4 to 9 years. **(E)** 10 to <18 years. **(F)** All age groups combined.

Descriptive data for the Gamma globulin fraction analysis are shown in **Table 3**. A significant positive association was observed between the Gamma and IgG values (**Figure 3**) for all age groups, separately and for the combined analysis. Gamma values were able to significantly explain part of the variance in IgG values in all groups: 0 to 5 months (88% **Figure 3A**), 6 to 11 months (88%, **Figure 3B**), 1 to 3 years old (91%, **Figure 3C**), 4 to 9 years old (92%, **Figure 3D**), 10 to < 18 years old (92%, **Figure 3E**). For the combined analysis of all samples, Gamma values explained 93% of the IgG values variance (**Figure 3F**).

**Table 3:** Characteristics of patients studied for the IgG *vs* Gamma fraction correlation^a^.

Age Group	Age (years)	IgG (g/L)	Gamma (g/L)	% males
1 to 5 mos (n = 23)	0,3 ± 0,1	4,4 ± 2,4	4,2 ± 2,7	47,6
6 to 11 mos (n = 56)	0,7 ± 0,1	5,4 ± 2	5,5 ± 1,9	45,3
1 yo to <4 yo (n = 364)	1,8 ± 0,8	8,4 ± 2,7	8,3 ± 2,5	54,1
4 yoto <10 yo (n = 442)	6,3 ± 1,7	10,1 ± 2,9	9,9 ± 2,9	51,6
10 yo to <18 yo (n = 330)	13 ± 2,3	11,3 ± 2,9	11,1 ± 3	55,6
All (n = 1215)	6,4 ± 4,78	9,6 ± 3,2	9,4 ± 3,2	53,2

a Data are presented as mean ± SD.

In analyzing discriminant Gamma cutoff values between patients with levels below the reference level for IgG, the predictive power was classified as exceptional (AUC from 0.963 to 1.00), with AUC being significant and acceptable accuracy for all age groups, except those younger than 1 yo (**Table 4**). For these groups, we could not establish discriminant Gamma cutoff values between patients with levels below the reference and normal for IgG because there were no patients with hypogammaglobulinemia.

**Table 4:** Gamma fraction values as a function of IgG levels.

Age Group	AUC	95% CI	p value	Gamma Cutoff value (g/L)^a^	Sensitivity	Specificity	Number of patients with IgG	
							Below reference values	Normal
1 to 5 mos (n = 21)	–	–	–	–	–	–	0	21
6 to 11 mos (n = 53)	–	–	–	–	–	–	0	53
1 yo to <4 yo (n = 370)	1	1 - 1	<0,001	3,55	1	0,997	6	364
4 yoto <10 yo (n = 441)	0,997	0,99 - 1	<0,001	5,65	1	0,979	12	429
10 yo to <18 yo (n = 329)	0,995	0,99 - 1	<0,001	6,2	1	0,981	11	318
All (n = 1214)	0,963	0,95 - 0,98	<0,001	6,15	1	0,878	29	1185

AUC, area under the curve. CI 95%, 95% confidence interval.

a Gamma values below which IgG levels were considered below reference.

The sensitivity values were 100% for all groups, and specificity varied between 97.9% and 99.7% in all age groups. Exceptional accuracy was also observed for the cutoff value obtained for the combined age groups (AUC = 0.963, P <0.001, sensitivity = 100%, and specificity = 87.8%, **Table 4**).

## Discussion

Primary and secondary antibody deficiencies are treatable conditions, frequently associated with diagnostic delays (8–10), leading to higher morbidity, mortality (13, 26), and overall costs of treatment (14).

This work shows that both CG and Gamma fraction can serve as correlates of IgG levels and could be used as screening methods for detecting antibody deficiency in children and adolescents. We found different cutoff values by age group, both for CG and Gamma, in keeping with the age dependent lower limit of the reference ranges for IgG (23). We demonstrate that CG or Gamma have a good to excellent correlation with IgG levels, independent of age group.

In previous studies, Jolles et al. (19) described CG as a screening method for adults in Wales, using the Architect Biuret method for total protein calculation and the bromocresol green method for albumin. The authors chose a cutoff value of CG < 18 g/L, which corresponded to a sensitivity of 0.82 and a specificity of 0.71 for an IgG < 3 g/l. Thereafter, Holding et al. (27) showed the results of an extensive screening program in England, using a rate biuret method or total protein and bromocresol purple for albumin. It is unclear if there were children or adolescents in the sample, but the authors chose a cutoff value for CG <18g/L, with a positive predictive value of 8.6% (7–11%) for IgG <3g/L. Pecoraro et al. (20), using the same methods as Jolles et al., chose a cutoff value of 19g/l to detect IgG levels below 6g/L, with a sensitivity of 70% and a specificity of 75%. This study was performed in adult Italian patients (>18 yo).

Assessment of the pediatric population and a different method for calculating total protein and albumin, namely serum protein electrophoresis, distinguish our study from those described above. In this regard, CG cutoff values were established for different age groups, ranging from 23.1 g/L in the 1 to 3 yo group to 24.8 g/L in the 4 to 9 yo group (see **Table 2** for details). This method’s accuracy also varied among the age groups, with sensitivity ranging from 90.9% in the 10 to <18 yo group to 100% in the 1 to 3 yo and 4 to 9 yo groups. Specificity also demonstrated a variation from 80.2% in the 4 to 9 yo group to 94.7% in the 10 to <18 yo group.

Gamma globulin fraction cutoff values to discriminate individuals with low IgG levels varied depending on the age groups (see **Table 4**). Interestingly, both the sensitivity and the specificity of this method for the whole group (100% and 87.8%, respectively) was slightly higher than those of CG (93.15% and 81.8%, respectively). However, the number of individuals identified below reference levels for IgG in the total sample was the same (29 individuals).

For children under 1-year-old, we evaluated the correlation between IgG *versus* CG in two groups, according to the age in months. Although the numbers of individuals were smaller compared to the whole group, all groups under one year had significant correlations between the parameters. Diagnosis of a primary antibody deficiency is less frequent in this population, as immunoglobulin levels in the newborn relate to the maternal-fetal transfer of antibodies. The maternal-fetal transfer of immunoglobulins is dependent on several factors, including maternal levels of total IgG and specific antibodies, gestational age, placental integrity, IgG subclass, and nature of antigen (28). The nadir for IgG levels occurs at three months of age, but transient hypogammaglobulinemia can persist because of a prolonged nadir (29). These factors make the diagnosis of hypogammaglobulinemia in infants <1 yo challenging. Furthermore, the small number of patients younger than 1 year in our study limited our ability to reach definitive conclusions.

IgG makes up around 75% of total serum immunoglobulins, with IgA levels usually 4 to 5 times, and IgM levels 7 to 10 times lower than IgG (30). Therefore, both the sensitivity and specificity of the test to detect IgA, IgM and IgG subclass deficiency is expected to be much lower. Specific antibody deficiencies cannot be detected using CG or Gamma fraction screening methods.

The aim of these tests is to screen for antibody deficiencies, in particular IgG as the major immunoglobulin class in blood, however, subsequent definitive diagnosis will require follow on tests, such as measurement of quantitative immunoglobulin levels, followed by B and T cell studies, functional antibody testing and/or genomic tests as appropriate.

Calculated globulin or Gamma fraction as screening tools for detecting IgG antibody deficiency fulfills all of the rules proposed by Wilson and Jungner (31) and most of the revised rules proposed by Dobrow et al. (32). The tests are low cost, readily available, and regularly performed to diagnose or follow-up other diseases or as routine/baseline testing. Our study indeed shows that CG or Gamma fraction were able to detect 29 cases of abnormal low IgG levels, 2.4% of the cases.

One limitation of our proof of principle study is the nature of the sample population (enriched for patients who sought Allergy/Immunology clinics and frequently presenting with a history of recurrent infections), which may lead to different levels of accuracy compared to other populations and the chosen cutoff values (23) may differ across settings. Another limitation (potentially an advantage), was the free patient choice of laboratories. This may impact the results, but is closer to real-life and clinical practice.

In conclusion, CG and Gamma fraction are simple screening methods for primary antibody deficiencies in children and adolescents. While this study did not include patients with secondary antibody deficiencies, CG screening detected secondary antibody deficiency in other studies (19). We have established age-dependent cutoff values for pediatric and adolescent patients using CG and Gamma fraction with the potential to decrease diagnostic delay, morbidity, mortality, and costs. In the future, it will be possible to introduce automated comments to prompt further investigation, such as IgG, IgM, and IgA determinations, when CG or Gamma fraction fall below the cutoff values, allowing earlier diagnosis and better outcome of antibody deficiency conditions. Further studies are needed in more general settings to evaluate the accuracy of these tests in a wider population.

## Data Availability Statement

The raw data supporting the conclusions of this article will be made available by the authors, without undue reservation.

## Ethics Statement

The studies involving human participants were reviewed and approved by The University of São Paulo and the Federal University of São Paulo Ethics Committees’ (approval number 3.340.392 and 3.499.511, respectively). Written informed consent to participate in this study was provided by the participants’ legal guardian/next of kin.

## Author Contributions

CP, data collection and manuscript writing. CA, patient selection and manuscript review. DS, patient selection and manuscript review. AC-N, study design, manuscript writing, and review. SJ study design and manuscript review. All authors contributed to the article and approved the submitted version.

## Funding

This work received research grants from Takeda and CSL Behring.

## Conflict of Interest

AC-N declare receiving speaker’s fees and participating in advisory boards for Takeda, CSL Behring, Novartis, AstraZeneca, GSK, and Sanofi Genzyme.

SJ has participated in advisory boards, trials, conferences, projects, and has been a speaker with CSL Behring, Takeda, Swedish Orphan Biovitrum, Biotest, Binding Site, Grifols, BPL, Octapharma, LFB, Pharming, GSK, Weatherden, Zarodex, Sanofi, and UCB Pharma.

The remaining authors declare that the research was conducted in the absence of any commercial or financial relationships that could be construed as a potential conflict of interest.

## Publisher’s Note

All claims expressed in this article are solely those of the authors and do not necessarily represent those of their affiliated organizations, or those of the publisher, the editors and the reviewers. Any product that may be evaluated in this article, or claim that may be made by its manufacturer, is not guaranteed or endorsed by the publisher.
